# Comparison of therapeutic effects of chemo-radiotherapy with neoadjuvant chemotherapy before radical surgery in patients with bulky cervical carcinoma (stage IB3 & IIA2)

**DOI:** 10.1186/s12885-021-08416-0

**Published:** 2021-06-05

**Authors:** Setareh Akhavan, Abbas Alibakhshi, Mahdieh Parsapoor, Abbas Alipour, Elahe Rezayof

**Affiliations:** 1 Gynecology Oncology Ward, Vali-e-Asr Hospital, Tehran University of Medical Sciences, Imam Khomeini Hospital Complex, Tohid Square, Tehran, 1419733141 Iran; 2 General Surgery Ward, Vali-e-Asr Hospital, Tehran University of Medical Sciences, Tehran, Iran; 3 Community Medicine Department, Medical Faculty, Thalassemia Research Center, Mazandaran University of Medical Sciences, Sari, Iran; 4 Vali-e-Asr Reproductive Health Research Center, Tehran University of Medical Sciences, Tehran, Iran

**Keywords:** Uterine cervical neoplasms, Chemotherapy, Bulky mass, Chemo-radiotherapy, Radical hysterectomy

## Abstract

**Background:**

Cervical cancer is one of the most common malignancies among women. Appropriate and timely treatment of these patients can reduce the complications and increase their survival. The objective of this study was to compare neoadjuvant chemotherapy plus radical hysterectomy (NACTRH) and chemo-radiotherapy (CRT) in patients with bulky cervical cancer (stage IB3 & IIA2).

**Material and methods:**

The medical records of patients with bulky cervical cancer (stage IB3 & IIA2) that received NACTRH or CRT between 2007 and 2017 were evaluated for therapeutic effects. Demographic characteristics, complications of chemo-radiotherapy and neoadjuvant chemotherapy, were collected in a researcher-made questionnaire. Our primary outcome was comparison of overall survival (OS), and disease-free survival (DFS) between two groups receiving NACTRH and CRT modalities.

**Results:**

One-hundred and twenty three patients were enrolled in the study. The median age and the proportion of patients with stage IIA2 were higher in the CRT group compared to the NACTRH group (*p* < 0.05). The medians (95% CI) OS were 3.64 (3.95–6.45) and 3.9 (3.53–4.27) years in the NACTRH and CRT groups, respectively (*P* = 0.003). There were 16 (34.8%) and 22 (43.1%) recurrences in the NACTRH and CRT group, respectively (*P* = 0.4). The median (95% CI) DFS was 4.5 (3.88–5.12) years in the NACTRH group and 3.6 (2.85–4.35) years in the CRT group (*P* = 0.004). The 3-year OS rate in NACTRH and CRT groups were 97 and 90% respectively. The 3-year DFS rate in NACTRH and CRT groups were 88 and 66% respectively.

**Conclusions:**

NACTRH is associated with a higher OS and DFS compared to CRT.

## Introduction

Cervical cancer is the third most common cancer among women and the fourth leading cause of death. It is the most common cancer in some developing countries [1]. Cervical cancer progresses slowly; therefore, it can be prevented by regular screening and planned gynecological examinations [2]. The masses larger than four centimeters are referred to as “bulky masses” (stages IB3 and IIA2) [3]. Although a clinical approach to cervical cancer is primarily dependent on the stage of the disease, the approach will be more complex if it is spread to adjacent areas such as lymph nodes and tissues around the pelvis [4].

Standard treatment includes radical hysterectomy or chemo-radiotherapy (CRT) in the early stages of the disease, which may cause sexual disorders and loss of fertility [4]. To improve the treatment outcomes in these stages, some other therapies have also been suggested, including the use of neoadjuvant chemotherapy before surgery, pre-surgery CRT, radiotherapy-sensitizing drugs alone or in combination with Cisplatin, and the use of biologic agents along with CRT [5].

Controlling the affected area in chemotherapy is a major problem in bulky tumors [5, 6]. Neoadjuvant chemotherapy before the initial radical surgery has some advantages, including reduced tumor volume, better tumor surgery, lower chance of disease recurrence, decreased need for adjuvant radiotherapy, and preservation of the ovaries [7, 8]. Chemotherapy prior to radiotherapy increases the patient’s survival by reducing the primary tumor size, increasing tumor vascularization, improving tumor radio-sensitivity, and eradication of micro-metastatic disease [9–11]. However, this approach may cause opposite results in cases of suboptimal dose of Cisplatin and prolonged intervals between therapy courses through increased regrowth of tumor cells resistant to chemo-radiotherapy and the prolongation of the treatment period [7].

Since cancer treatment success, improved patient survival, and decreased relapse rate have always been a major concern, the present study was conducted to compare the therapeutic effects and survival rate of chemo-radiotherapy and neoadjuvant chemotherapy prior to radical surgery in patients with cervical cancer (stage IB3 & IIA2).

## Materials and methods

### Samples

This study was carried out in the gynecology-oncology Departments of Vali-e-Asr Hospital, Imam Khomeini Hospital Complex, and a tertiary referral center for gynecological problems in Tehran, Iran. In this retrospective cohort study, the medical records of patients with bulky cervical cancer who underwent chemo-radiotherapy or neoadjuvant chemotherapy plus radical surgery (NACTRH) for the first time were investigated. Cases with previous peripheral neuropathy, other malignancies, those with a platelet count below 100,000/ml, WBC count below 4000/ml, bilirubin levels above 1.5 mg/dl, serum creatinine levels above 1.2, a positive history of systemic diseases (including diabetes, chronic liver disease, chronic kidney disease and immunodeficiency), and the patients that were impossible to access were excluded from the study. All patients’ staging was done clinically. Abdominopelvic MRI was performed for all of patients who underwent NACTRH. Patients who had parametrial or lymph node involvement in imaging excluded from our study. For those patients who received concurrent chemoradiation abdominopelvic CT scan was performed before RT planning. Patients who had lymph node involvement in imaging were not the target of our study.

Patients with bulky cervical cancer who were candidates for neoadjuvant chemotherapy before surgery received 3 courses of chemotherapy with cisplatin 80 mg/m^2^ and paclitaxel 60 mg/m^2^. The interval between each course was considered 10 days. The surgery was performed 3–4 weeks after the last course of chemotherapy. All patients tolerated this chemotherapy regimen without any severe complications. Candidate patients for chemoradiation underwent pelvic External Beam Radiotherapy (EBRT) at approximately 45 Gy with concurrent weekly administration of 40 mg/m^2^ cisplatin. Then, they received brachytherapy at 30 to 40 Gy. All treatments were completed within 7 to 8 weeks.

### Data collection method

One-hundred and twenty-three (123) medical records of eligible patients who were first diagnosed with bulky mass cervical cancer and received NACTRH or CRT from 2007 to 2017 were extracted. Past medical history; cigarette, hookah and alcohol use; family history of cervical cancer; disease status, including the date of diagnosis, stage (bulky), grade (1–3), and tumor size; and history of systemic diseases (hypertension, hyperlipidemia, etc.) were recorded in a researcher-made questionnaire. After recording the data, the subjects were evaluated for therapeutic effects, including overall survival (OS), disease-free survival (DFS), mortality rate, and morbidity rate after treatment. Data were collected using the patients’ medical records and telephone contacts.

### Data analysis

The characteristics of the participants and the tumor were grouped according to the treatment state.

Chi-square was used to compare the categorical variables between the treatment groups. T-test and Mann Whitney U test were applied to compare continuous variables such as age and gravidity. The overall survival (OS) and disease free survival (DFS) outcome were estimated according to the Kaplan–Meier method. Cox proportional hazards regression models were used to estimate the relative likelihood of DFS and OS with various factors in both univariate and multivariate analyses. The results are presented as hazard ratios (HRs) with 95% confidence interval (Cis). *P* values of less than 0.05 were considered significant. Statistical analysis was performed using the SPSS 16.0 (version 16.0; SPSS Inc., Chicago, IL, USA).

## Results

### Clinical and pathological characteristics

In this study, 123 cervical cancer patients with a median age of 50 (41–57) years were investigated. Seventy two and fifty-one patients received NACTRH and CRT, respectively. Twenty six of patients who received NACTRH underwent radiation after surgery. To prevent any bias, 26 patient who were indicated for radiotherapy after NACTRH, were excluded from survival study. Of all patients who received neoadjuvant chemotherapy (46), 6 cases showed drop of haemoglobin. Blood transfusion was performed for 4 patients pre-operation. They underwent surgery 4 weeks after the end of chemotherapy. The notable surgical complications in the NACTRH group were seen in 6 cases including: fever (6), wound infection (4) and urinary problems (2). In the CRT group the, 12 cases showed complications. The most important complications were proctitis (3), skin burns (5), vaginal fibrosis (1), sexual problems (10), and vesicovaginal fistula (1).

The median follow-up was 3.5 (3.1–4) years. Other characteristics of the patients, based on treatment status, are shown in Table 1. As shown in Table 1, the median age and the proportion of patients with stage IIA2 were higher in the CRT group compared to the NACTRH group (*p* < 0.05).

**Table 1:** Demographic and baseline characteristics of the study cohort divided into patients that received neoadjuvant chemotherapy and radical hysterectomy (NACTRH) or chemo-radiotherapy (CRT)

	Group		
	NACTRH (***n*** = 46)	CRT (***n*** = 51)	***P*** value
**Age, year, Median (SD)**	46.41 (10.8)	53.06 (11.02)	0.004
**Gravid, Median (IQR)**	3 (1–3)	2 (1–3)	0.93
**Fallow up time, year, Median (IQR)**	3.85 (3.48–4.3)	3.2 (2.8–3.7)	< 0.001
**Stage, n (%)**			
**IB3**	29 (63)	21 (41.2)	0.03
**IIA2**	17 (37)	30 (58.8)	
**Surgery complications, n (%)**	6 (13)	–	NA
**Chemotherapy complications, n (%)**	6 (13)	10 (19.6)	0.38
**Chemo-radiotherapy complications, n (%)**	–	12 (23.5)	NA

### Mortality rate and overall survival

**Fig. 1:**
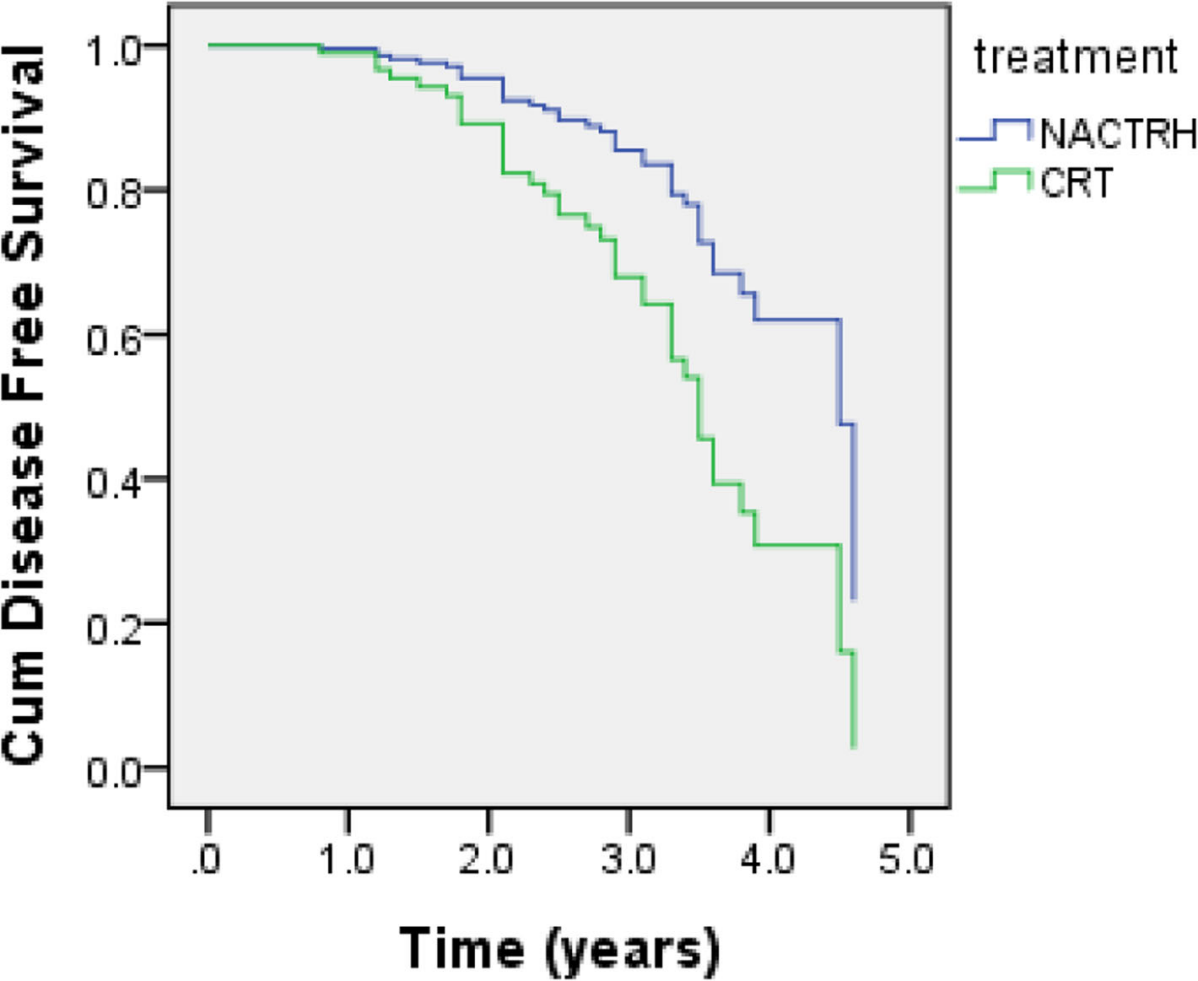
Survival curves of disease-free time using Cox regression method

The mortality rate was 0.11 in 100 person-years (0.08–0.15). There were 14 (30.4%) and 20 (39.2%) deaths in the NACTRH and CRT group, respectively (*P* = 0.37). The medians (95% CI) OS were 3.64 (3.95–6.45) years in the NACTRH and 3.9 (3.53–4.27) years in the CRT group (*P* = 0.003) (Fig. 1). Univariate analysis suggested that age and therapy state were significantly associated with OS (Table 2). According to the multivariate Cox proportional hazards models, as shown in Table 2, independent prognostic factors for OS were age [HR = 1.06 (95% CI: 1.04–1.12)], and CRT vs NACTRH [HR = 2.38 (95% CI: 1.13–5.41)]. The 3-year OS rate in NACTRH and CRT groups were 97 and 90% respectively.

**Table 2:** Multivariate Analysis of time to death related to cervical cancer

	Value or Characteristic Compared	Crude Hazards Ratio	Adjusted Hazards Ratio	***P*** value
**Age**	1 year increasing	1.08 (1.05–1.12)	1.08 (1.04–1.12)	< 0.001
**Treatment**				
**NACTRH**	–	1	1	–
**CRT**	CRT vs. NACTRH	2.94 (1.4–6.2)	2.47 (1.13–5.41)	0.02
**Stage**	IB2 vs. IIA2	1.45 (0.73–2.88)	0.75 (0.42–1.87)	0.75

### Recurrence rate and disease- free survival

The recurrence rate was 0.17 in 100 person-years (0.13–0.22). There were 16 (34.8%) and 22 (43.1%) recurrences in the NACTRH and CRT groups, respectively (*P* = 0.4). In the both groups, first signs of recurrence were in the pelvic cavity. The main site of recurrence was vaginal cuff. After vaginal cuff, pelvic lymph node was the second common site of recurrence.

**Fig. 2:**
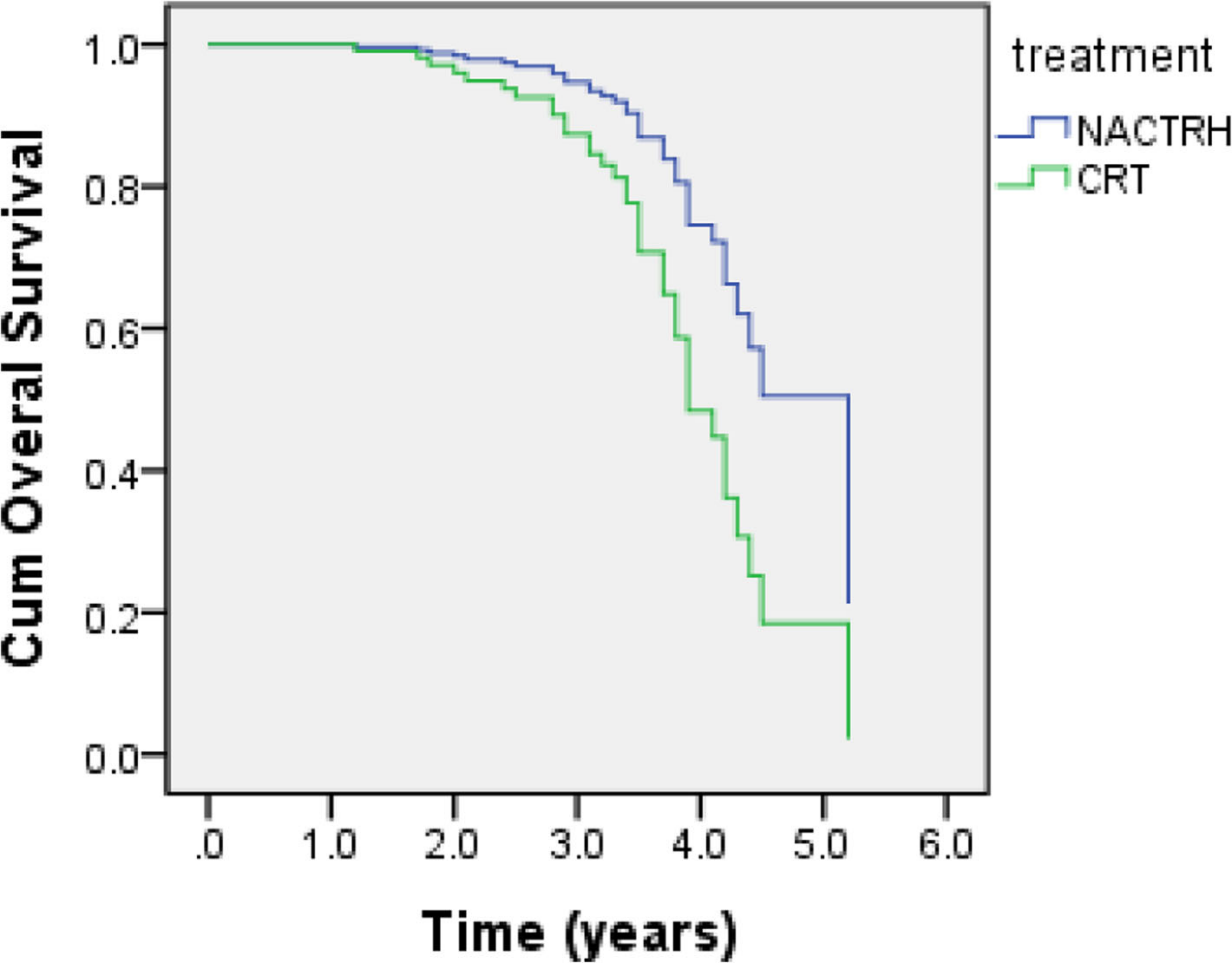
Survival curves of overall survival using Cox regression method

The median (95% CI) DFS was 4.5 (3.88–5.12) years in the NACTRH and 3.6 (2.85–4.35) years in the CRT group (*P* = 0.004) (Fig. 2). Univariate analysis showed that age and treatment status had a significant association with DFS (Table 3). According to the multivariate Cox proportional hazards models, as shown in Table 2, independent prognostic factors for DFS were age [HR = 1.06 (95% CI: 1.02–1.1)] and CRT vs NACTRH [HR = 3.48 (95%CI: 1.46–8.31)]. The 3-year DFS rate in NACTRH and CRT groups were 88 and 66% respectively. Twenty-six patients (56.52%) required radiotherapy after NACTRH according to pathology results. The most important cause of radiotherapy in these patients was lymph node involvement which was seen in 10 patients. The second most common cause was parametrial involvement.

**Table 3:** Multivariate Analysis of time to disease recurrence related to cervical cancer

	Value or Characteristic Compared	Crude Hazards Ratio	Adjusted Hazards Ratio	***P*** value
**Age**	1 year increasing	1.03 (1.02–1.1)	1.06 (1.02–1.1)	< 0.001
**Treatment**				
**NACTRH**	–	1	1	–
**CRT**	CRT vs. NACTRH	3.5 (1.52–8.05)	3.48 (1.46–8.31)	0.03
**Stage**	IB2 vs. IIA2	1.21 (0.57–2.57)	0.85 (0.39–1.87)	0.63

## Discussion

According to data from studies investigating the survival and recurrence rates of cervical cancer, radical hysterectomy is a good alternative in the early stages of bulky masses; however, NACTRH can be helpful depending on the patient’s condition [12, 13].

In the present study, the median age and the proportion of patients with stage IIA2 were significantly higher in the CRT group compared to the NACTRH group. Similarly, in a study by Qin et al., the mean age of the patients that underwent NACTRH was higher compared to the patients who received radiotherapy after hysterectomy; however, this difference was not statistically significant [14].

Several studies have investigated the survival rate, disease-free survival rate, and recurrence rate of cervical cancer using different therapeutic approaches [12–18]. In 2019, Zhao et al. conducted a review study to compare the overall survival, disease-free survival, and local/distant recurrence between neoadjuvant chemotherapy prior to surgery and surgery alone and found no difference between the two groups in 13 studies including 2158 cervical cancer patients. In an analysis of 8 studies including 1544 patients with stages IB2 (at present IB3)–IIB, the overall survival rate, the extent of distant metastasis, and disease relapse decreased significantly in the group that received neoadjuvant chemotherapy prior to radical hysterectomy [9]. Other studies are required to determine the effect of pre-surgery neoadjuvant chemotherapy on the overall survival and disease-free survival in different groups of patients with cervical cancer.

According to the present findings, the disease-free survival rate was longer in patients who received NACTRH compared to CRT. The results of similar studies also show that the disease-free survival rate is significantly prolonged in the NACTRH group [10, 14].

In our study the overall survival and disease free survival were higher in the NACTRH group than the CRT group. On the contrary, Marchetti et al. by a meta-analysis showed that the effects of both modalities (NACTRH and CRT) were the same regarding overall survival [19]. It seems this difference between the results may relate to our small sample size or early stage of disease in our patients.

In the present study, 56.52% of the patients in the NACTRH group needed radiotherapy after pathological examination. Some studies have found that regardless of the type of treatment, a large pathological tumor size and an immediate complication contribute to a worse outcome^16^. The results of a study conducted by Landoni et al. showed that CRT and surgery were the preferred treatment options for stage Ib-IIa cervical cancer that had the same efficacy but different rates of complications and disabilities. Significant complications were seen in 28% of cases that underwent surgery and 14% of the cases that received CRT [11]. Another study also indicated that simultaneous use of CRT and surgery increased complications [20].

Very few studies have investigated the effect of chemo-radiotherapy alone on the survival rate of cervical cancer patients with bulky masses. Radiotherapy is often used as adjuvant after radical hysterectomy; however, it has been reported that adding chemotherapy to definitive radiotherapy in patients with stage IB1 or stage IIA1 cervical cancer is associated with an improvement in OS [21]. In the present study, none of the patients underwent radical hysterectomy after chemo-radiotherapy. Univariate analysis showed that age and treatment type had significant association with disease-free and overall survival. However, a study found no significant differences in 5-year disease-free and overall survival between radical surgery patients and those receiving radiotherapy [22].

According to the present results, the recurrence rate was 34.8 and 43.1% in the NACTRH and CRT group, respectively. In line with this finding, Wan et al. reported that the recurrent rate was 26.5% in the NACT group and 90.9% in patients that underwent upfront surgery [23].

The death rate was lower in the NACTRH group compared to the CRT group. Based on the results of a meta-analysis, in line with the present findings, NACTRH caused a reduction of 35%in the risk of death [24]. In addition, older age at diagnosis and treatment played an important role as the risk factor of both overall survival and disease-free survival.

The main limitation of the present study was our small sample size. This limitation may relate to diagnosis of disease in the advanced stages that frequently is observed among patients in the low/middle countries [25].

## Conclusion

As we know, the success of cancer treatment, improved patient survival, and reduced relapse rate have always been a major concern. Our results showed the advantages of NACTRH before the initial radical surgery. The rate of relapse in patients with CRT was higher compared to NACTRH; therefore, the disease-free survival rate was higher in the NACTRH group. The rate of disease recurrence and the risk of death were lower in the patients receiving NACTRH.

### Limitations

The limitations of the study are the small number of patients and its retrospectiveness.

Further prospective studies with more number of patients are needed to confirm these findings.

## Data Availability

Data sharing not applicable to this article as no datasets were generated or analysed during the current study.

## Abbreviations

NACTRH: Neoadjuvant chemotherapy plus radical hysterectomy
CRT: Chemo-radiotherapy
OS: Overall survival
DFS: Disease-free survival
TUMS: Tehran University of Medical Sciences
